# PKM2-mediated metabolic reprogramming of microglia in neuroinflammation

**DOI:** 10.1038/s41420-025-02453-5

**Published:** 2025-04-06

**Authors:** Qi Zhang, Sha-Sha Wang, Zhao Zhang, Shi-Feng Chu

**Affiliations:** 1 Basic medicine college, China Three Gorges University, Yichang, China; 2 State Key Laboratory of Bioactive Substances and Functions of Natural Medicines, Institute of Materia Medica & Neuroscience Center, Chinese Academy of Medical Sciences and Peking Union Medical College, Beijing, China

**Keywords:** Neuroimmunology, Stroke

## Abstract

Microglia, the resident immune cells of the central nervous system, undergo metabolic reprogramming during neuroinflammation, playing a crucial role in the pathogenesis of neurological disorders such as Parkinson’s disease. This review focuses on Pyruvate Kinase M2 (PKM2), a key glycolytic enzyme, and its impact on microglial metabolic reprogramming and subsequent neuroinflammation. We explore the regulatory mechanisms governing PKM2 activity, its influence on microglial activation and immune responses, and its contribution to the progression of various neurological diseases. Finally, we highlight the therapeutic potential of targeting PKM2 as a novel strategy for treating neuroinflammation-driven neurological disorders. This review provides insights into the molecular mechanisms of PKM2 in neuroinflammation, aiming to inform the development of future therapeutic interventions.

## Facts

- Neuroinflammation plays an important role in the pathogenesis of nervous system diseases. Currently, anti-neuroinflammatory therapies have become a key strategy for treating these diseases.
- Microglia metabolic reprogramming regulates neuroinflammatory responses through various pathways.
- PKM2, a key molecule in glycolysis, has been recognized for its role in exacerbating neuroinflammation by regulating microglial metabolic reprogramming.

## Open questions

- How does PKM2 affect microglia function and immune response?
- What are the molecular mechanisms by which PKM2 regulates microglia metabolic reprogramming?
- What role does PKM2 play in the pathogenesis of various common neurological diseases?
- What is the therapeutic potential of targeting PKM2 to reduce neuroinflammation?

## Introduction

Metabolic reprogramming, the shift from oxidative phosphorylation to aerobic glycolysis to meet energy demands under external stimuli, facilitates cell proliferation and survival [1]. This adaptation, observed in cancer, aging, inflammation, immune responses, and stress-related disorders [2, 3], confers both stress resistance and new functionalities. Neuroinflammation, a critical mechanism in neurological diseases like Alzheimer’s disease [4], Parkinson’s disease [5], and ischemic stroke [6], is characterized by microglial activation and aggregation around neurons, altering the immune microenvironment through increased cytokine/chemokine secretion and vascular permeability [7]. These changes can disrupt neuronal function and intercellular communication. As the resident immune cells in the central nervous system (CNS), microglia can adapt their metabolic pathways to the changes of brain microenvironment, modulating their immune function and neuroinflammatory responses. Pyruvate kinase M2 (PKM2) overexpression induces microglial activation [8], driving metabolic reprogramming towards aerobic glycolysis. Therefore, the interaction between microglia and PKM2 in neuroinflammation has garnered much attention. Consequently, the interplay between PKM2 and microglia in neuroinflammation has become a focal point of research. Modulating PKM2 expression to control microglial metabolism and suppress neuroinflammation represents a promising therapeutic strategy. Further investigation into the metabolic reprogramming mechanisms of microglia and PKM2 may yield effective strategies to regulate this metabolic shift and improve disease outcomes.

## Overview of PKM2

**Fig. 1:**
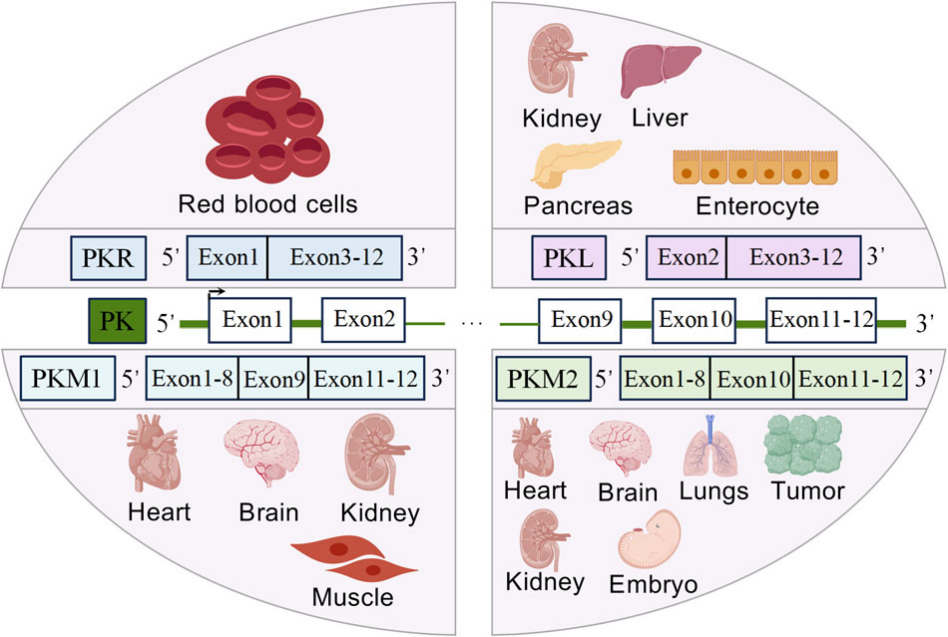
Expression of pyruvate kinase isoforms. Mammals express four pyruvate kinase isoforms: PKL, PKR, PKM1, and PKM2, encoded by two PK genes. One gene encodes PKL and PKR, sharing exons 3-12. PKR transcripts retain exon 1 and are predominantly expressed in red blood cells. PKL transcripts retain exon 2 and are primarily expressed in the liver, kidney, pancreas, and intestine. The other gene encodes PKM1 and PKM2, sharing exons 1-8 and 11-12. PKM1 transcripts retain exon 9 and are expressed in differentiated tissues such as the heart, brain, kidney, and muscle. PKM2 transcripts retain exon 10 and are expressed in brain, lung, liver, kidney, heart, embryonic tissues, and tumor cells. PKM2 expression is significantly upregulated during inflammation.

Glycolysis, the anaerobic breakdown of glucose to lactate, culminates with the rate-limiting enzyme pyruvate kinase (PK) [9], which catalyzes the conversion of phosphoenolpyruvate (PEP) and ADP to pyruvate and ATP [10]. Mammals express four PK isoforms: pyruvate kinase M1 (PKM1), PKM2, pyruvate kinase L (PKL) and pyruvate kinase R (PKR) [11]. Two PK genes encode these isoforms: one encodes PKL (expressed in liver, kidney, pancreas, and intestine) and PKR (expressed in red blood cells). The other gene encodes PKM1 and PKM2. PKM1 is found in differentiated tissues like the heart, brain, kidney, and muscle, contributing to muscle energy metabolism. PKM2 is widely expressed, including in the brain, liver, lung, kidney, heart, islet β-cells, neurons, embryos, and tumor cells [12] (Fig. 1).

The human PKM gene, located at 15q22, spans ~ 32 kb and comprises 12 exons and 11 introns, with multiple promoters and splice sites [13]. PKM1 and PKM2 arise from alternative splicing: both share exons 1-8 and 11-12, differing only in exons 9 and 10. The final PKM2 mRNA retains exon 10, while PKM1 retains exon 9 [14]. This results in 22 amino acid differences within the 56 amino acids encoded by these exons [15]. Despite sharing metabolic enzymes and kinase activity, PKM1 and PKM2 exhibit distinct active conformations [16]. PKM1 exists solely as a highly active tetramer, whereas PKM2 exists as monomers, dimers, and tetramers [17]. Under physiological conditions, cytoplasmic and mitochondrial PKM2 exist as a tetramer [18], exhibiting high activity and PEP affinity, efficiently catalyzing glycolysis and producing ATP and metabolites essential for cellular function [19]. Hypoxia or altered metabolic states can promote the accumulation of substrates and shift metabolic pathways, favoring the dimeric form of PKM2 [20]. Dimeric PKM2 can modulate signaling pathways and epigenetic modifications to support cell metabolism, proliferation, and inflammation [21, 22]. Notably, PKM2 predominantly exists as a dimer in human monocytes and macrophages, but as a monomer in mouse macrophages [23], highlighting species-specific regulatory mechanisms and the diverse biological functions of PKM2. Thus, PKM2’s conformational state plays a crucial role in regulating macrophage/microglial metabolic function and disease pathogenesis.

## PKM2 and the immune status of microglia

### Microglia activation and migration

Microglia, the resident macrophages of the CNS [24], polarize to the pro-inflammatory M1 state following CNS injury or disease, eliminating pathogens and clearing cellular debris. During later stages of injury or chronic inflammation, microglia transition to the anti-inflammatory M2 state, promoting tissue repair and resolving inflammation. However, excessive microglial activation can exacerbate neuroinflammation, damage the blood-brain barrier (BBB), and impair neurological recovery. Therefore, maintaining balanced microglial activation is crucial for CNS health.

PKM2 is implicated in microglial polarization, with high expression correlating with increased metabolic activity and energy demand, driving microglial activation and metabolic shifts [25]. NADPH oxidase 4 (NOX4) induces M1 polarization via the PKM2 pathway in microglia [26]. PKM2 knockdown, which blocks glycolysis, prevents LPS-induced M1 polarization in macrophages [27]. The neuropsychiatric systemic lupus erythematosus (NPSLE) murine model serves as an experimental system to recapitulate depression, anxiety, cognitive impairment, and psychosis-like symptoms observed in NPSLE patients. Notably, elevated PKM2 expression in hippocampal microglia within this model is associated with upregulation of β-catenin and its downstream targets c-Myc and Cyclin-D1. In vitro, PKM2 enhanced microglial activation and synaptic phagocytosis via the β-catenin pathway. Conversely, inhibiting microglial PKM2 ameliorated cognitive impairment and brain damage in NPSLE mice [28]. PKM2 also contributes to microglial activation in traumatic brain injury (TBI), with increased expression during acute and subacute phases, and PKM2 inhibition improves cognitive function [29]. Triclosan (TCS) promotes microglial activation and inflammatory cytokine release by increasing PKM2 expression in BV-2 cells, further supporting PKM2’s role in driving neuroinflammation through metabolic reprogramming [30]. These studies collectively implicate PKM2 in microglial activation. Beyond direct effects on microglia, PKM2 influences microglia-neuron/astrocyte interactions, regulating immune responses and maintaining metabolic homeostasis and energy supply to protect the CNS [14]. This suggests context-dependent roles for PKM2 in cellular metabolism. Further investigation into PKM2’s role in different cell types and microglial polarization will provide valuable insights into its function in neuroinflammation and microglial responses to injury and inflammation.

**Fig. 2:**
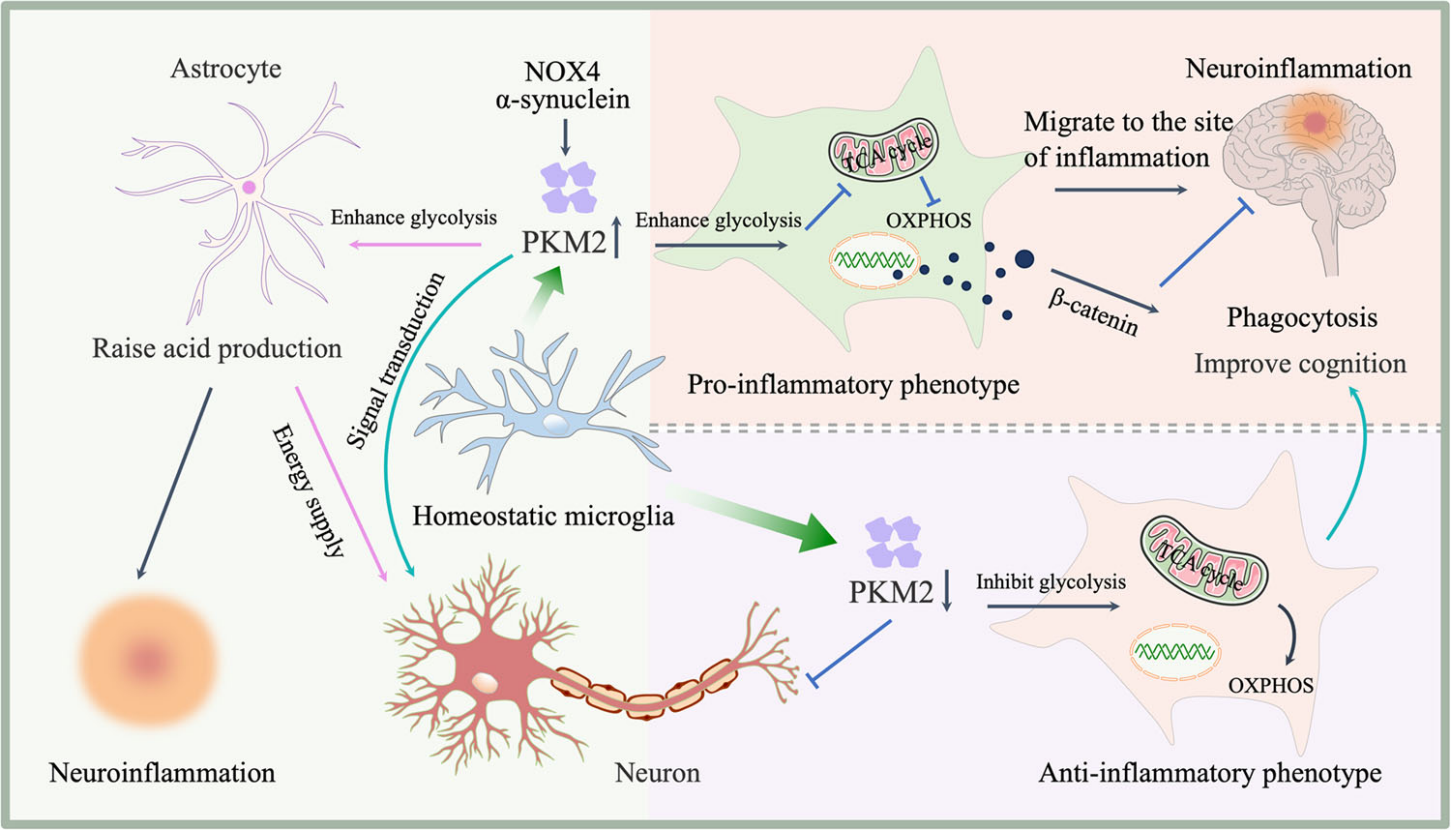
PKM2 and microglial immune status. During neuroinflammation, upregulated PKM2 in microglia drives metabolic reprogramming, inducing pro-inflammatory (M1) polarization and chemotaxis. Enhanced glycolytic acid production in astrocytes and neurons exhibits dual effects: promoting neuronal activity at physiological levels while exacerbating inflammatory cascades when overaccumulated. PKM2 inhibition reverses this metabolic shift by augmenting oxidative phosphorylation, thereby triggering an anti-inflammatory (M2) phenotypic transition that confers neuroprotection.

PKM2 not only influences microglial polarization but also promotes migration to inflammatory sites. Following spinal cord injury, α-synuclein (αSyn) interacts with PKM2 to drive microglial metabolic reprogramming and enhance migration [5]. As a key regulator of cellular physiology and pathology, PKM2 is increasingly recognized as a central player in CNS inflammatory diseases (Fig. 2).

### PKM2 in other cells

Beyond its role in microglia, PKM2 is also important in neurons and astrocytes. Neuronal PKM2, localized in cell bodies, supports high levels of aerobic glycolysis, protecting against oxidative stress. Mice lacking PKM2 exhibit increased oxidative damage and dopaminergic neuron loss [31]. As shown on the left side of Fig. 2, normal astrocytic PKM2 expression is essential for maintaining astrocyte energy supply and neuronal excitability. However, PKM2 overexpression in astrocytes promotes excessive aerobic glycolysis, exacerbating inflammatory neuropathic pain. These findings suggest that while PKM2 participates in metabolism in both neurons and astrocytes, its role in neurons focuses on synaptic plasticity and signal transduction, whereas in astrocytes, it primarily supports and protects neurons.

## Related molecules involved in PKM2 regulation

### HIF-1α

Hypoxia-inducible factor-1α (HIF-1α), a transcription factor activated under hypoxic conditions, promotes the metabolic shift from oxidative phosphorylation to glycolysis. HIF-1α directly upregulates PKM2 expression by binding to its promoter region, enhancing microglial glycolytic capacity and potentially playing a neuroprotective role. Conversely, upregulated PKM2 can translocate to the nucleus as a low-activity dimer, acting as a transcriptional coactivator by phosphorylating HIF-1α. The resulting PKM2-HIF-1α complex binds to the IL-1β promoter, inducing pro-inflammatory cytokine secretion and exacerbating inflammation [32]. HIF-1α-enhanced PKM2-mediated microglial inflammation also involves the NLRP3 inflammasome and NF-κB p65 [33, 34]. Thus, HIF-1α and PKM2 interactions regulate microglial energy metabolism and survival, enabling adaptation to adverse environments but also potentially driving excessive activation and inflammation. Further research exploring their roles in different neuropathological states could inform therapeutic strategies targeting this mechanism.

### STAT3

Signal transducer and activator of transcription 3 (STAT3), activated by cytokine signaling, regulates genes involved in inflammation, cell proliferation, and survival. TCS increases PKM2 dimerization and nuclear translocation in prefrontal cortex microglia, where nuclear PKM2 promotes STAT3 phosphorylation at Tyr705, inducing microglial activation and cytokine release [35]. This highlights the detrimental role of PKM2-regulated STAT3 phosphorylation in TCS-induced behavioral changes. Nuclear PKM2 can phosphorylate STAT3, promoting IL-6 and IL-1β release and downstream inflammatory responses, while alleviating oxidative stress and inflammation in macrophages during coronary atherosclerosis [36]. Further research could explore the roles of STAT3 and PKM2 in cerebrovascular diseases. Chronic constriction injury (CCI) induces neuropathic pain and cognitive decline in rats. PKM2 inhibition may attenuate CCI-induced neuropathic pain and inflammation through modulation of ERK and STAT3 signaling pathways [37]. Blocking the STAT3 pathway reduces PKM2-induced TNF-α and IL-1β expression, suggesting that STAT3 mediates PKM2’s pro-inflammatory effects and implicating the NF-κB-PKM2-STAT3 axis in regulating inflammatory cytokine release [38].

### Pyk2

Proline-rich tyrosine kinase 2 (Pyk2), a non-receptor tyrosine kinase, activates downstream signaling pathways through substrate phosphorylation. Pyk2 activation promotes microglial inflammatory responses. PKM2 activates Pyk2 by enhancing macrophage glycolysis, which in turn activates downstream TLR4, TLR7, and TLR9 pathways involved in inflammation and autoimmunity [39].

### HDACs

Histone deacetylases (HDACs), particularly HDAC7, are key inflammatory drivers in innate immune cells, linking metabolic changes to inflammation. They connect TLR-triggered aerobic glycolysis with macrophage activation. The HDAC7-PKM2 complex acts as a signaling hub, with HDAC7 deacetylating PKM2 at lysine 433 to activate its pro-inflammatory function. Disrupting this interaction suppresses inflammation in vitro and in vivo, highlighting the importance of class IIa HDACs and PKM2 in regulating metabolic and inflammatory interplay during immune responses [40].

### Acetyl-histone H3K9

In hypoxic or inflammation-induced microglial models, PKM2 expression, nuclear translocation, and acetyl-histone H3K9 expression are upregulated, suggesting a positive correlation. Nuclear PKM2 mediates ischemia-induced microglial polarization through interaction with acetyl-histone H3K9. Inhibiting PKM2 nuclear translocation reduces ischemia-induced inflammation and promotes neuronal survival [41].

### ATF2

Activating transcription factor 2 (ATF2) is involved in cell proliferation, apoptosis, and stress responses. Nuclear PKM2 interacts with ATF2 in microglia, promoting glycolysis and thermogenesis [42]. Inhibiting PKM2 nuclear translocation reduces ATF2 phosphorylation and microglial activation. Similarly, ATF2 knockdown reduces LPS-induced microglial activation. These results suggest that PKM2 promotes microglial metabolic reprogramming and neuroinflammation via ATF2.

**Fig. 3:**
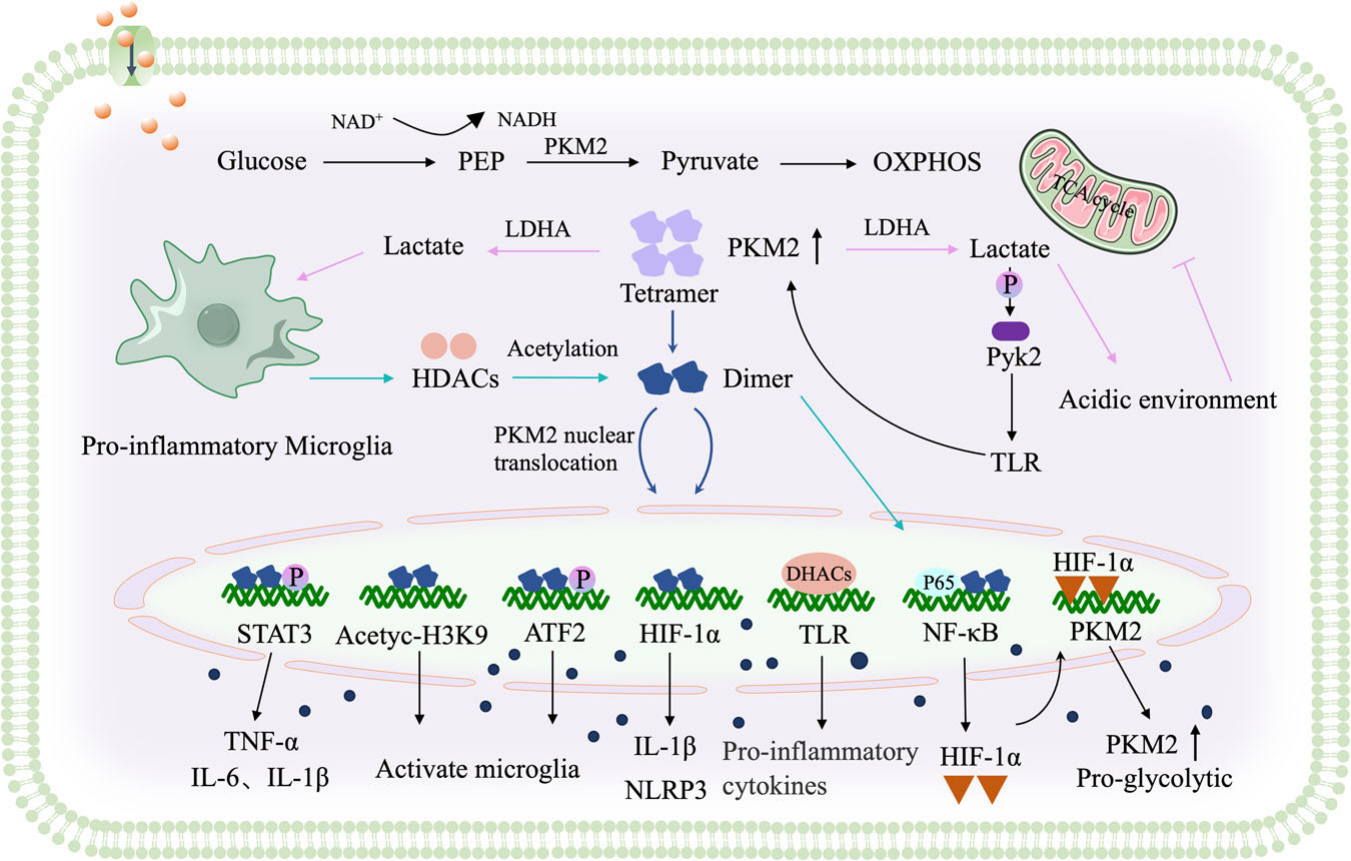
Regulation of PKM2. Under physiological conditions, cytoplasmic PKM2 exists as a tetramer. Upon microglia activation, PKM2 dissociates into dimers and translocates to the nucleus to regulate gene expression. HIF-1α directly upregulates PKM2 expression and its nuclear translocation. In the nucleus, PKM2 phosphorylates HIF-1α, forming a complex that promotes IL-1β secretion. Additionally, PKM2 phosphorylates STAT3 to enhance the release of IL-6/IL-1β, thereby exacerbating the inflammatory response. Moreover, PKM2 interacts with signaling pathways such as ATF2, Pyk2, β-catenin, and mTOR to regulate its own expression, glycolysis, and microglial polarization within pro-inflammatory environments.

Related molecules involved in PKM2 regulation are listed in Fig. 3.

## PKM2-mediated microglial metabolic reprogramming in neurological diseases

### PKM2 and ischemic stroke

Ischemic stroke (IS) is a leading cause of death and disability worldwide. The resulting ischemic and hypoxic environment disrupts brain cell metabolism and exacerbates damage, making understanding its underlying mechanisms crucial. PKM2 is implicated in post-stroke inflammation and neural repair. Inhibiting PKM2 nuclear translocation reduces microglial polarization to a pro-inflammatory phenotype. PKM2 is upregulated after cerebral ischemia-reperfusion injury (CI/RI) and exacerbates neuroinflammation via the TLR4/MyD88/TRAF6 pathway [43]. PKM2 knockout reduces infarct volume, neurological dysfunction, neuronal injury, and inflammation induced by oxygen-glucose deprivation/reperfusion (OGD/R). Neonatal hypoxic-ischemic encephalopathy (HIE)-induced neuronal injury leads to severe motor and sensory dysfunction. Notably, PKM2 may exacerbate neurological deficits by triggering post-HIE neuronal apoptosis through a mechanism mediated by p-AKT inactivation [44]. PKM2 deletion in bone marrow cells limits peripheral neutrophil inflammatory responses and reduces neutrophil extracellular traps after I/R, suggesting that PKM2 promotes neutrophil hyperactivation and exacerbates post-stroke inflammation [45]. These findings indicate that elevated PKM2 levels negatively impact IS and that PKM2 antagonists may offer neuroprotection.

While PKM2 appears detrimental in microglia after IS, it may exert protective effects on neurons and angiogenesis. Loss of PKM2 in astrocytes and neurons, or reduced lactate supply due to PKM2 inhibition by MiR-143 and MiR-19a, exacerbates neuronal death after CI/RI [46]. Recombinant PKM2 administration during acute and subacute IS phases may exert neuroprotective effects via STAT3 and FAK signaling, contributing to neurovascular regeneration and functional recovery [47]. PKM2 may also exert anti-inflammatory and anti-oxidative effects in post-stroke depression (PSD) rats by activating the VEGF-mediated MAPK/ERK pathway [48]. Promoting vascular repair and regeneration after IS is critical, as excessive inflammation disrupts the BBB. PKM2 promotes VEGF production in vascular endothelial cells, inhibits NF-κB and downstream targets, and protects the vascular barrier [49]. JMJD8 may interact with PKM2 to enhance endothelial cell glycolysis and vascular sprouting [50]. These results suggest a significant role for PKM2 in angiogenesis, and further research is needed to verify its potential protective effects on the BBB after IS. Thus, PKM2 represents a promising therapeutic target for post-stroke neurological recovery.

### PKM2 and Alzheimer’s disease

Uncontrolled microglial activation and neuroinflammation are implicated in Alzheimer’s disease (AD) pathogenesis [51]. Given the strong link between inflammation and glucose metabolism in AD, PKM2 has emerged as a potential therapeutic target. Proteomic analysis of post-mortem AD brains reveals elevated PKM2 levels, a finding corroborated in AD mouse models [52]. Interestingly, this PKM2 elevation is associated with glial cell activation. Recent studies have shown that microglia-specific PKM2 knockout ameliorates Aβ pathology in AD mice. Blocking PKM2 disrupts the glycolytic/H4K12la/PKM2 loop, reducing microglial activation and improving spatial learning and memory [4]. Furthermore, PKM2 also promotes Aβ production by mediating hypoxia-induced γ-secretase activation via APH-1, exacerbating cognitive impairment [53]. These results implicate PKM2 in AD pathogenesis.

PKM2 inhibition may also protect neurons in AD. Inhibiting PKM2 nuclear translocation in human fibroblast-induced neurons restores neuronal metabolism, reverses AD-specific gene expression, and reduces apoptosis [54]. Conversely, PKM2 inhibition by MiR-326 can induce endoplasmic reticulum stress and oxidative stress, exacerbating neuronal apoptosis and AD progression [15]. These findings suggest a complex role for PKM2 in regulating neuronal metabolism and repair, offering potential therapeutic avenues for AD.

### PKM2 and Parkinson’s disease

αSyn, a key driver of Parkinson’s disease (PD) pathogenesis, induces microglial glycolysis via PKM2 phosphorylation. PKM2 inhibition significantly reduces αSyn-induced microglial migration and neuroinflammation [5]. Oxidative stress-induced mitochondrial dysfunction in dopaminergic neurons also contributes to PD. Enhancing PKM2 ubiquitination and enzymatic activity can ameliorate this dysfunction, providing neuroprotection [38, 55]. Astrocytic dopamine D2 receptors regulate glutathione (GSH) biosynthesis, crucial in neurodegenerative diseases [56], by promoting PKM2 dimerization, nuclear translocation, and Nrf2 activation, ultimately enhancing GSH production and neuroprotection [57]. Blood PKM2 levels inversely correlate with PD rating scale scores in high-risk individuals [58], implicating PKM2 dysregulation in PD. In diabetic patients, PKM2-mediated neuronal aerobic glycolysis increases PD risk [59], suggesting context-dependent roles for PKM2 in PD.

### PKM2 and other neurological diseases

PKM2 is implicated in various neurological diseases, including epilepsy, glioma, neuropathic pain, and axon metabolism. In glioma, PKM2 promotes tumor cell proliferation and metastasis. PKM2-generated lactate is essential for maintaining peripheral axonal metabolism and signaling. In epilepsy, enhancing PKM2 expression through deubiquitination promotes astrocytic lactate production, improving local energy supply and reducing seizure frequency. While PKM2 overexpression in microglia can exacerbate neuroinflammation, it plays a neuroprotective role in neurons and astrocytes, highlighting the different roles of PKM2 in different diseases.

## Modulators of PKM2 in inflammatory diseases

### PKM2 modulators for neuroinflammation

PKM2 has emerged as a promising therapeutic target for neuroinflammatory diseases. Panax notoginseng saponins exert anti-inflammatory effects in cardiovascular and cerebrovascular diseases by inhibiting microglial PKM2 expression and downregulating the HIF-1α/PKM2/STAT3 pathway [60]. Tetrahydroxy stilbene glucoside (TSG) promotes an anti-inflammatory microglial phenotype by inhibiting PKM2 nuclear translocation, offering neuroprotection in IS [61]. 3,5-Bis-trifluoromethylphenyl-substituted BPM29 downregulates PKM2 expression in BV2 microglia, inhibiting NLRP3 inflammasome formation and promoting an anti-inflammatory phenotype, suggesting its potential for treating demyelinating diseases [8]. CM292, an acetamide-based iNOS inhibitor, reduces PKM2 nuclear translocation, increases mitochondrial membrane potential and oxygen consumption, attenuates glycolysis, and inhibits LPS-induced BV2 microglial activation [62]. These findings support PKM2’s role in microglial phenotype switching. Benzoxepane derivatives exert anti-neuroinflammatory effects in vitro and in vivo by inhibiting PKM2-mediated glycolysis and NLRP3 activation [63]. Offering a potentially safer alternative to shikonin. The cannabidiol derivative CIAC001, a PKM2 activator, exerts anti-neuroinflammatory effects and mitigates morphine withdrawal symptoms by promoting PKM2 tetramerization and inhibiting its nuclear translocation. Currently in the preclinical research phase, this compound demonstrates therapeutic potential for addressing opioid addiction [64]. Dimethylaminomicheliolide Fumarate (ACT-001), another PKM activator, is undergoing phase II clinical trials for glioma, CNS tumors, and optic neuritis [65]. While PKM2-targeted drug development predominantly centers on cancer metabolic modulation, preliminary investigations have begun exploring its therapeutic potential in neurodegenerative and inflammatory diseases. We have summarized the PKM2 modulators and their specific mechanisms in a Table 1.

**Table 1:** Modulators of PKM2 and their mechanisms.

Compound	Signaling pathway	Effects	Refs
PKM2 modulators targeting microglia			
Panax notoginseng	HIF-1α/PKM2/STAT3	Reducing PKM2 expression	[60]
BPM 29	PKM2/NLRP3	Reducing PKM2 expression	[8]
Tetrahydroxy stilbene glucoside		Promoting PKM2 tetramerization and inhibiting nuclear translocation	[61]
CIAC001		Promoting PKM2 tetramerization and inhibiting nuclear translocation	[64]
ACT-001	STAT3/NF-κB/	PKM activator	[65]
CM292		Inhibiting of PKM2 nuclear translocation	[62]
Benzoxepane derivatives	PKM2/NLRP3	Inhibiting PKM2-mediated glycolysis	[63]
PKM2 modulators targeting macrophages			
DASA-58	PKM2 /HIF-1α/ IL-1β	Promoting PKM2 tetramerization and reducing lactate secretion	[76]
Annexin A5	ASP101, LEU104, ARG106/PKM2/Y105	Promoting PKM2 tetramer formation	[67]
ML-265		Promoting PKM2 tetramer formation and increasing PKM2 enzymatic activity	[77]
TEPP-46	PKM2 /HIF-1α/ IL-1β	Promoting PKM2 tetramer formation and increasing enzymatic activity	[76]
Sfn	PKM2 /HIF-1α/ IL-1β	Promoting PKM2 tetramer formation and increasing enzymatic activity	[78]
Norisoboldine	PKM2/HIF-1α/PGC-1α	Promoting PKM2 tetramer formation and increasing enzymatic activity	[70]
Shikonin		Decreasing PKM2 enzymatic activity	[79]
Celastrol	Binding with residue CYS31	Decreasing PKM2 enzymatic activity	[80]
IncRNA HITT	HIF-1α/PKM2	Reducing lactate secretion	[81]
Deoxyelephantopin		Reducing glycolysis	[82]
Compound 3 K		Triggering PKM2 tetramer disruption	[67]
Emodin	PKM2/Nrf2/ARE	Promoting dimer formation and nuclear translocation	[68]
SIRT5	PKM2 /HIF-1α/ IL-1β	Promoting dimer formation and nuclear translocation	[83]
Iminostilbene	PKM2/HIF1α/STAT3	Reducing PKM2 expression	[72]
Melittin	Akt/mTOR/PKM2/HIF-1α	Reducing PKM2 expression	[84]
Paraquat		Reducing PKM2 expression	[85]
Plumbagin	NOX4/PKM2	Reducing PKM2 expression	[86]
ω-alkynyl arachidonic acid	KM2/HIF-1α/iNOS	Reducing PKM2 expression and nuclear translocation	[71]
Lycium barbarum polysaccharide		Reducing PKM2 expression and enhancing ubiquitination	[66]
SYK	PKM2/NLRP3/IL-1β	Promoting nuclear translocation of PKM2	[87]
Iridin		Inhibiting of PKM2 phosphorylation	[27]
ITA	HSP90/ITA/PKM2/Bcl2	Promoting mitochondrial translocation of PKM2	[88]
HDACs	PKM2/TLR	Reducing glycolysis	[40]
DRAM1	Binding to PKM2	Increasing the expression of PKM2 in the serosa	[89]
Cynaroside	PKM2/HIF-1α	Polarization from pro-inflammatory M1 type to anti-inflammatory M2 type	[69]
SENP3	HIF-1α/PKM2	Inhibiting pro-inflammatory M1 polarization	[90]
Aucklandiae radix	PKM2/NF-κB/NLRP3	Inhibiting inflammatory phenotype	[91]
Tubeimoside-1	PKM2/Caspase-3 /GSDME	Activating caspase-3 and cleaving gasdermin E through the inhibition of PKM2	[92]
Digoxin	KM2/HIF-1α	Inhibiting PKM2 targeting HIF-1α transactivation	[93]

### PKM2 modulators for other inflammations

Natural bioactive compounds targeting PKM2 have shown promise in modulating inflammation. The first is the role in macrophage polarization. Lycium barbarum polysaccharide (LBP) reduces LPS-induced inflammation by downregulating PKM2 expression through enhanced ubiquitination, inhibiting glycolysis and pro-inflammatory macrophage polarization [66]. Annexin A5 promotes M2 polarization in liver macrophages by targeting PKM2 tetramerization [67]. Emodin, known for its antioxidant and neuroprotective effects, promotes PKM2 dimerization and nuclear translocation, activating the Nrf2/ARE pathway and protecting PC12 cells from oxidative damage [68]. Cynaroside targets the PKM2/HIF-1α axis, inhibiting PKM2 nuclear translocation and preventing pro-inflammatory macrophage polarization [69]. Norisoboldine regulates the PKM2/HIF-1α/PGC-1α pathway, promoting M2 polarization and alleviating sepsis-induced acute lung injury [70]. In addition, ω-Alkyl arachidonic acid modulates the PKM2/HIF-1α/iNOS link, promoting anti-inflammatory macrophage polarization in acute myocardial infarction [71]. Iminostilbene (ISB) reduces PKM2 expression, HIF-1α expression, and STAT3 phosphorylation, inhibiting macrophage activation and alleviating myocardial I/R injury [72]. Further research is needed to determine whether ISB has similar effects in CI/RI. These findings highlight the importance of PKM2-mediated macrophage polarization in immune-related diseases and offer potential therapeutic strategies using natural compounds. However, research on PKM2 modulators in neuroinflammation remains limited. Given PKM2’s established roles in inflammation [73], myocardial infarction [74], and lung injury [75], further investigation into its mechanisms in neuroinflammation is crucial for developing targeted therapies.

## Summary and prospect

PKM2, a recognized target in cancer cell metabolic reprogramming, is also emerging as a promising therapeutic target for inflammatory diseases. Microglial PKM2 expression and activity during neuroinflammation are subject to multi-level regulation, encompassing subcellular localization, gene transcription, post-translational modification, and mitochondrial function. Growing research on PKM2 in neurological diseases is elucidating its diverse roles in various biological processes. While several PKM2 modulators show promise in neuroinflammatory disease models in vitro, further in vivo and clinical validation is needed to assess their efficacy, safety, and biocompatibility. Similarly, clinical trials are lacking for activators and inhibitors of related neuroinflammatory targets. Natural compounds, with their multi-target and multi-pathway therapeutic properties, have demonstrated efficacy in treating neuroinflammation. Their structural complexity, diverse biological activities, lower development costs, and improved safety profiles make them attractive candidates for PKM2 modulation. These compounds hold significant potential as a source for novel PKM2 modulators for neuroinflammation therapy.

## Data Availability

Data sharing is not applicable to this article as no datasets were generated or analyzed during the current study. All data generated or analyzed during this study are included in this published article or available from the corresponding author on reasonable request.
